# Impact of the volume-based procurement for intraocular lenses combined with the diagnosis-related group’s payment reform on hospitalization costs and expenditure structure among cataract patients: an interrupted time series analysis

**DOI:** 10.3389/fpubh.2026.1905462

**Published:** 2026-09-10

**Authors:** Lisha Zhou, Pengfei Zhou, Lin Shu, Kuanyu Xu, Qiang Lu, Feng Xiao, Haibo Liu, Xiaoguang Yang, Yunming Li

**Affiliations:** 1 School of Public Health, Southwest Medical University, Luzhou, China; 2 Office of Medical Information and Data, Medical Support Center, The General Hospital of Western Theater Command, PLA, Chengdu, China; 3 Department of Health Economics, The General Hospital of Western Theater Command, PLA, Chengdu, China; 4 Department of Information, Medical Support Center, The General Hospital of Western Theater Command, PLA, Chengdu, China; 5 Department of Medical Engineering, Medical Support Center, The General Hospital of Western Theater Command, PLA, Chengdu, China

**Keywords:** cataract, China, DRG payment, hospitalization cost, interrupted time series analysis, volume-based procurement

## Abstract

**Background:**

Cataract surgery is one of the most resource-intensive procedures in China, with intraocular lens (IOL) costs accounting for a substantial proportion of total hospitalization expenditure. To address persistently high prices of high-value medical consumables, our hospital implemented the Beijing-Tianjin-Hebei “3 + N” regional alliance procurement for IOLs in January 2022, followed by DRG payment reform in January 2024. However, the synergistic effects of these two sequential policies on cataract surgery costs—particularly on both cost levels and expenditure composition—have not been systematically evaluated.

**Methods:**

We conducted a retrospective analysis of 8,458 cataract patients who underwent IOL implantation at a tertiary hospital in western China from January 2021 to December 2025. A multiple-phase interrupted time series model was used, with monthly medians of costs and cost proportions as outcomes. Two intervention points were defined: IOL-VBP implementation and DRG payment reform.

**Results:**

Immediately after VBP, total hospitalization cost, material cost, and surgical cost dropped significantly, though a rebound followed with total and material costs shifting to a monthly increase. Specifically, the median total hospitalization cost decreased from 27,403.68 CNY before the reform to 7,214.21 CNY after VBP, and further to 5,516.60 CNY after DRG—a cumulative reduction of 79.9%; the median material cost declined from 11,119.44 CNY to 4,382.92 CNY after VBP, and then to 2,741.10 CNY after DRG—a reduction of 75.4%. DRG reversed the rebound: total and material costs declined continuously, and the material cost proportion continued to fall. The surgical cost proportion rose from 27.44% to 35.35%, although the median surgical fee remained unchanged—a denominator effect driven by sharper declines in diagnostic and other cost components. Surgical volume nearly tripled over the study period, yet annual analysis using mean costs revealed that real per-case surgical revenue decreased, indicating that total revenue growth was entirely volume-driven.

**Conclusion:**

The VBP-DRG combination effectively contained costs and shifted expenditure structure toward services at the relative level. However, this structural improvement did not translate into higher absolute physician compensation. To address this, future reforms should consider dynamic adjustments of DRG weights and service prices to align cost containment with fair recognition of medical professionals’ skills.

## 1 Introduction

In China, medical services are predominantly delivered by public hospitals (1). Since the economic reforms of the 1980s, government subsidies to public hospitals have declined from approximately 40% in the early 1980s to around 10% in the 1990s (2, 3). To compensate for this reduction while maintaining affordable services, the government allowed hospitals to generate revenue through a 15% markup on drugs and a 10% markup on medical consumables under a fee-for-service system (1, 2). These incentives, coupled with performance-based bonuses linked to revenue generation, created a distorted compensation structure that encouraged excessive use of drugs and consumables, and fueled the rapid growth of healthcare costs (4).

To address these challenges, the Chinese government launched a new round of healthcare reform in 2009, with a primary goal of enhancing the public welfare character of public hospitals (5, 6). A key reform was the abolition of the drug markup policy, completed by September 2017. However, this reform led to unintended consequences: hospitals shifted their profit-seeking behavior from drugs to high-value medical consumables and diagnostic tests. By 2019, the proportion of drug revenue in public hospitals had decreased substantially from pre-reform levels—from approximately 40% in 2012 to around 27% in 2019—while the proportion of revenue from medical consumables increased during the same period (7, 8). Subsequently, by the end of 2019, the government also abolished the markup on medical consumables in all public hospitals, further reshaping the incentive structure.

To fundamentally address this distorted incentive structure and close remaining loopholes, the Chinese government introduced two complementary reforms. First, the Volume-Based Procurement (VBP) policy—a major pharmaceutical and healthcare reform initiative led by the National Healthcare Security Administration (NHSA) operating on the principle of “state organization, alliance-based procurement, and platform operation”—was expanded from drugs to high-value medical consumables (9). The policy aims to reduce procurement prices of drugs and medical consumables through collective bargaining power while ensuring supply quality (10, 11). In November 2020, the first national VBP for coronary stents was conducted, reducing prices from approximately 13,000 CNY to 700 CNY (12). The policy was first applied to intraocular lenses (IOLs) in 2020, when the Beijing-Tianjin-Hebei “3 + N” nine-province alliance conducted the first round of centralized procurement, achieving an average price reduction of 46.4% from historical procurement levels (13). In January 2022, the alliance—now expanded to 23 provinces, with 14 participating in the IOL procurement—conducted a second round of procurement, achieving an average price of 2,347 CNY per IOL, representing a further reduction of 16.91% from the 2020 procurement cycle (14). The cumulative reduction from pre-procurement levels reached approximately 55.5%, and the policy was rolled out across participating provinces in early 2022. Second, the Diagnosis-Related Groups (DRG) payment reform, which originated in the United States in the 1970s, was introduced to constrain hospital behavior from the payment endpoint (15). Based on the principle of “similar clinical characteristics and similar resource consumption,” DRG classifies inpatient cases by diagnosis, treatment, complications, and age, assigning a standardized payment amount to each group (16). In 2019, the NHSA launched DRG pilots in 30 cities, and by the end of 2021, all 30 DRG pilot cities had implemented actual payment (11). The reform was expanded nationwide in 2024. Evaluations of early implementation have demonstrated its cost-containment effects: a quasi-experimental study using data from 53 hospitals in Beijing found that DRG-based prepayment for patients with neurologic disorders was associated with a 12.6% reduction in total costs per case and a decrease of 0.96 days in length of stay (17). These two policies are theoretically complementary—VBP reduces procurement costs from the pricing source, while DRG imposes financial discipline from the payment endpoint—and may generate a synergistic “1 + 1 > 2” effect. Cataract surgery, one of the most common procedures involving high-value consumables, serves as an ideal case for examining the real-world impact of these policy combinations.

Cataract, an age-related opacification of the crystalline lens, is a leading cause of blindness globally (18, 19). As one of the countries with the most severe aging populations in Asia, China has experienced a rapid increase in cataract prevalence: there were approximately 19.78 million cases in 2021—a 248.08% increase from 1990—and the number is projected to exceed 240 million by 2050 (20, 21). The primary treatment is surgical removal of the opacified lens with IOL implantation, with consumable costs often exceeding 50% of total surgical expenditure (22).

While previous studies have separately confirmed that VBP can reduce consumable prices and DRG can lower hospitalization costs, several critical gaps remain (23, 24). First, research on the synergistic effect of these two policies in the same patient population is scarce, with most studies focusing on a single policy. When both policies are implemented simultaneously, hospitals may partially offset the overall cost-containment effect through behavioral adjustments—such as increasing the volume of consumables used, switching to non-procured products, or raising other service fees—a possibility that has yet to be fully revealed. Second, most existing studies have focused on per-case costs and cost proportions, while overlooking changes in total hospitalization costs—a critical gap given that surgical volumes have substantially increased over time. Third, the absolute changes in surgical costs and their implications for physicians’ labor value compensation remain underexplored. To address these gaps, we employ a multi-phase interrupted time series design, which allows us to disentangle the independent and interactive effects of the two sequential policies. Therefore, this study aims to evaluate the synergistic effect of IOL-VBP and DRG payment reform on cataract patients undergoing IOL implantation, examining median costs by category, cost composition, total annual costs, and absolute changes in surgical fees. Using a multi-phase interrupted time series design with real-world data from a tertiary hospital in western China (2021–2025), we assess the immediate and long-term effects of the combined policies, providing empirical evidence for optimizing the synergistic mechanism between health insurance payment and medical consumables governance.

## 2 Methods

### 2.1 Study setting and research team

This study was conducted at the General Hospital of the Western Theater Command, a tertiary Grade-A general hospital located in Chengdu, in western China. The data were obtained from the hospital’s case management and hospital information system (HIS). The hospital serves as a regional medical center for the surrounding provinces and has been actively participating in national pilot programs for both volume-based procurement (VBP) and Diagnosis-Related Groups (DRG) payment reform, making it an ideal setting for evaluating the synergistic effects of these policies.

This research was carried out by a multidisciplinary team based within the hospital, comprising members from the Department of Health Economics, the Information Office, the Information and Data Center, and the Department of Medical Engineering. This internal collaborative structure provides us with unique institutional access and practical insights that are typically unavailable to external research teams. Specifically: (1) the Department of Medical Engineering maintains detailed records of high-value consumable procurement, usage, and inventory management, which allows us to precisely track the supply-side changes driven by VBP; (2) the Information Office oversees the operation and maintenance of the hospital information system (HIS), ensuring the stability, security, and proper functioning of the data infrastructure; (3) the Information and Data Center is responsible for data extraction, integration, and quality assurance, enabling us to construct a comprehensive, longitudinal dataset covering the full study period; and (4) the Department of Health Economics provides expertise in cost-accounting methodologies, payment reform analysis, and health policy evaluation. This collaborative, hospital-embedded perspective enables us to interpret our quantitative findings not merely as statistical associations, but as reflections of actual operational and financial dynamics within a real-world healthcare delivery system.

### 2.2 Data source and study sample

The data for this study were obtained from the case management and HIS of the same hospital. The study sample included all 8,458 civilian cataract patients who underwent IOL implantation surgery at this hospital over a 60-month period, from January 2021 to December 2025. All patients in the sample had a primary or secondary diagnosis of cataract (ICD-10 codes with the first three characters: H25–H26). In our dataset, we recorded whether the surgery was unilateral or bilateral. Among the 8,458 included patients, 8,434 (99.716%) underwent unilateral cataract surgery and 24 (0.284%) underwent bilateral procedures. Given the negligible proportion of bilateral cases, the main analysis combined both types. A sensitivity analysis excluding bilateral cases yielded consistent results (Supplementary Tables 1 and 2). We collected detailed hospitalization cost information, along with demographic and sociological characteristics for each patient, to support our analysis. Based on the information released by the Chengdu Municipal Bureau of Statistics, we selected the healthcare category from the Consumer Price Index (CPI) for residents of Chengdu. To eliminate the influence of inflation and ensure comparability across years, all hospitalization costs were converted to constant 2021 prices using the following formula: Adjusted cost = Nominal cost × (100/CPI). The CPI coefficients for 2022, 2023, 2024, and 2025 were 99.7, 100.2, 100.2, and 100.9, respectively, with the 2021 CPI serving as the baseline (2021 CPI = 100).

### 2.3 Variables

The total hospitalization cost was divided into six categories: pharmacy cost, material cost, surgery cost, treatment cost, diagnostic cost (including laboratory test cost and examination cost), and other cost (including nursing cost, bed cost, blood cost, traditional Chinese medicine cost, anesthesia cost, and other cost) (25). Each of the above cost categories and their corresponding proportions of the total cost were treated as dependent variables. The interrupted time series model was employed to analyze the level changes and trend changes before and after the implementation of the policies (26).

### 2.4 Study design and models

This study aims to describe the effects of the three time periods of the VBP and the DRG payment reform implementation on the hospitalization costs of cataract patients. After normality tests, both the total hospitalization cost and the structure of hospitalization costs were found to have a skewed distribution. Therefore, the median and the interquartile range (IQR) were used for descriptive statistics and the interrupted time series modeling. The changing trends of the total cost and its components from January 2021 to December 2025 are displayed by line graphs. In these line graphs, each data point represents the monthly median of the respective cost indicator or cost proportion, aggregated from all individual patient records within that calendar month. For the baseline characteristics, continuous variables following a normal distribution were described using the mean and standard deviation, while those with a non-normal distribution were described using the median and interquartile range (IQR). Categorical variables were described using frequencies and percentages. Comparisons among the three time periods were conducted using the Kruskal-Wallis test for continuous variables or the chi-square test for categorical variables.

Using January 1, 2022 (the implementation date of the VBP for intraocular lenses) and January 1, 2024 (the implementation date of the DRG payment reform) as the intervention time points, the study period was divided into three time periods: Time Period 1 (pre-reform) spanned from January 2021 to December 2021, during which 1,001 patients were included; Time Period 2 (post-VBP and pre-DRG) spanned from January 2022 to December 2023, during which 2,981 patients were included; and Time Period 3 (post-DRG) spanned from January 2024 to December 2025, during which 4,476 patients were included. The VBP for intraocular lenses was defined as Reform 1, and the DRG payment reform was defined as Reform 2 (Table 1).

**Table 1:** Correspondence between the two policies and the three periods.

Stage	Policy
Time Period 1 (2021.1–2021.12)	Before reform
Time Period 2 (2022.1–2023.12)	After the VBP (Reform1)
Time Period 3 (2024.1–2025.12)	After the DRG payment reform (Reform2)

The interrupted time series (ITS) design evaluates the actual effect of an intervention by assessing the changes in the level and trend of outcome indicators before and after the intervention. It can not only evaluate immediate effects but also observe the long-term developmental trends, and has been widely applied in the field of health policy evaluation (26, 27). Based on these characteristics, this study employs an interrupted time series model to evaluate the immediate and long-term effects of the two policies. The independent variable is time (*t*), measured in months. The dependent variables are the median of monthly total hospitalization cost, the median of each cost category, and the median of the proportion of each cost category in the total cost. The ITS model is specified as shown in Equation (1) below:


$$\begin{array}{l} Y_{t}=\beta_{0}+\beta_{1}\times t+\beta_{2}\times \text{refor}m_{1}\times \beta_{3}\times \text{timeafte}r_{1}\\ +\beta_{4}\times \text{refor}m_{2}+\beta_{5}\times \text{timeafte}r_{2}+\varepsilon_{t} \end{array}$$ (1)

In this model, “*Y_t_*” represents the dependent variable, denoting the median or proportion of various costs for each month; “*t*” is a time variable, representing the months, taking values of 0, 1, 2, 3. 59, with 0 corresponding to January 2021; “*reform*_1_” is a dichotomous dummy variable indicating whether the VBP intervention was implemented, taking the value of 0 for the pre-intervention period and 1 for the post-intervention period; “*reform*_2_” is a dichotomous dummy variable indicating whether the DRG payment policy intervention was implemented, taking the value of 0 for the pre-intervention period and 1 for the post-intervention period. “*time_after*_1_” and “*time_after*_2_” are time variables counting from the intervention point, used to estimate the trend change in the outcome indicator after policy implementation (i.e., the difference between the post-intervention slope and the pre-intervention slope).

Where $\;\beta_{0}$ represents the baseline level before the implementation of the policies; $\beta_{1}$ represents the secular trend before the policy intervention, i.e., the slope in the pre-intervention period; $\beta_{2}$ and $\beta_{4}$ represent the immediate level changes at the time of the VBP intervention and the DRG payment policy intervention, respectively; $\beta_{3}$ and $\beta_{5}$ represent the sustained changes in trend following the implementation of the VBP and the DRG payment reform, i.e., the difference between the post-intervention slope and the pre-intervention slope for the VBP and the DRG payment reform, respectively; $\beta_{1}$ + $\beta_{3}$ represents the slope after the implementation of the VBP; similarly, $\beta_{1}$ + $\beta_{3}$ + $\beta_{5}$ represents the slope after the implementation of the DRG payment policy; and $\varepsilon_{t}$ represents the error term.

In the Results section, to avoid confusion between trend changes and absolute trends, we report both the slope change coefficients (*β₃* and *β₅*) and the actual phase-specific slopes. The actual slopes were estimated as linear combinations of the regression coefficients—*β₁* for the pre-VBP period, *β₁ + β₃* for the post-VBP period, and *β₁ + β₃ + β₅* for the post-DRG period.

In addition to the descriptive and ITS analyses based on median per-case costs, we also calculated annual total hospitalization costs for each period by summing all individual costs. The mean per-case cost was used in this calculation (as total = mean × volume) because aggregate measures require additive properties, which medians do not possess.

This study used R software (version 4.5.2) for descriptive statistics and the interrupted time series analysis. All statistical tests were two-sided, and a significance level of α = 0.05 was used throughout the study. A segmented regression model was fitted based on ordinary least squares (OLS) regression, and significance tests of the regression coefficients were performed to determine the statistical significance of the trend changes in hospitalization costs and the proportions of each cost category before and after the VBP and the DRG payment reform. After fitting the segmented regression model, the Breusch-Pagan (BP) test and the Durbin-Watson (D-W) test were employed to assess the model for heteroscedasticity and autocorrelation. If the *p*-value of the BP test was more than 0.05, the residuals were considered to be homoscedastic; otherwise, heteroscedasticity was present, and robust standard errors (HC1) were used for correction (26). If the *p*-value of the Durbin-Watson test was more than 0.05, no autocorrelation was detected in the residuals; otherwise, autocorrelation was present, and Newey-West standard errors were used for correction (28). Finally, the adjusted R-squared (adjusted *R*^2^) was used to evaluate the goodness-of-fit of the model.

### 2.5 Ethics statement

This study was a retrospective analysis of fully anonymized data extracted from the hospital information system of the General Hospital of the Western Theater Command. Prior to data extraction, the hospital’s information management department irreversibly removed all personal identifiers, including names, national identification numbers, home addresses, phone numbers, and hospital admission numbers, ensuring that no individual patient could be identified. The research team signed a formal data confidentiality agreement, pledging that the data would be used exclusively for this study, with no external disclosure, unauthorized copying, or commercial use. All analyses were performed within the hospital’s encrypted internal network, in strict compliance with the hospital’s data security regulations. According to China’s national regulation “Measures for the Ethical Review of Biomedical Research Involving Human Subjects” and the institutional policy of the General Hospital of the Western Theater Command, retrospective studies using only anonymized data that pose no physical harm or risk to individuals are exempt from formal ethical committee approval. The study was conducted in accordance with the Declaration of Helsinki.

## 3 Results

### 3.1 General characteristics

Regarding the age composition, the proportions of patients aged 65 years and older in the three time periods were 61.1, 61.7, and 60.8%, respectively, and the difference in the overall age composition was not statistically significant (*χ^2^* = 0.552, *p* = 0.759). In terms of gender distribution, the proportions of female patients in the three time periods were 56.2, 57.3, and 59.7%, respectively, with a corresponding decrease in the proportion of male patients; however, the difference in the overall gender composition was not statistically significant (*χ^2^* = 5.878, *p* = 0.052) (Table 2).

**Table 2:** Basic information of patients with cataracts in each stage [*n* (%)].

	Total *n* = 8,458	Time Period 1 *n* = 1,001	Time Period 2 *n* = 2,981	Time Period 3 *n* = 4,476	*χ^2^*	*p*
Age (year)					0.552	0.759
<65	3,284	389 (38.9)	1,142 (38.3)	1753 (39.2)		
≥65	5,174	612 (61.1)	1839 (61.7)	2,723 (60.8)		
Gender					5.878	0.052
Male	3,495	460 (43.8)	1,298 (42.7)	1827 (40.3)		
Female	4,963	590 (56.2)	1745 (57.3)	2,708 (59.7)		

### 3.2 Median of hospitalization costs

**Figure 1:**
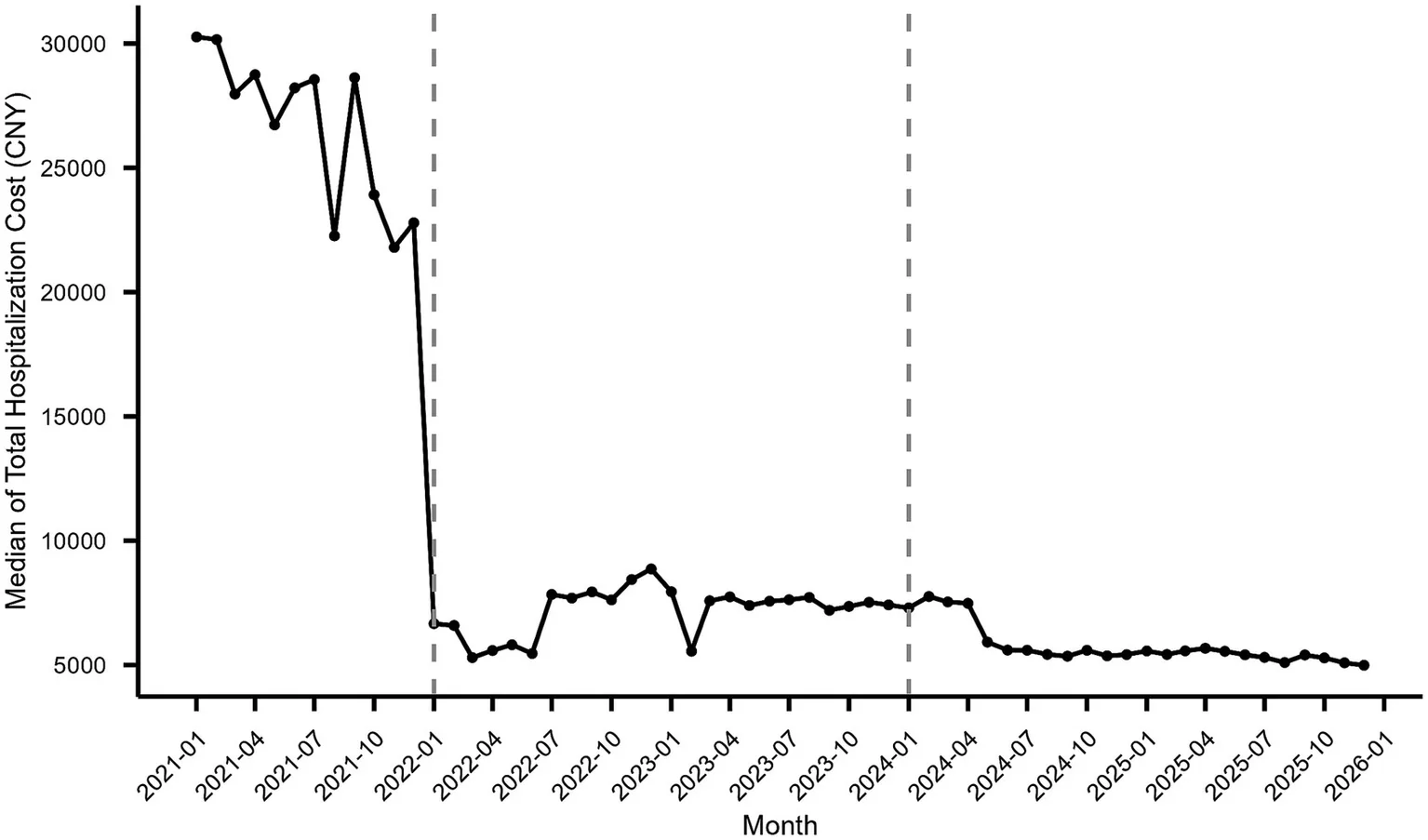
The time trend of median total hospitalization cost. Vertical dashed lines indicate the implementation dates of the VBP (January 2022) and DRG (January 2024) policies. Each point represents the monthly median aggregated from all individual patient records within that calendar month.

**Figure 2:**
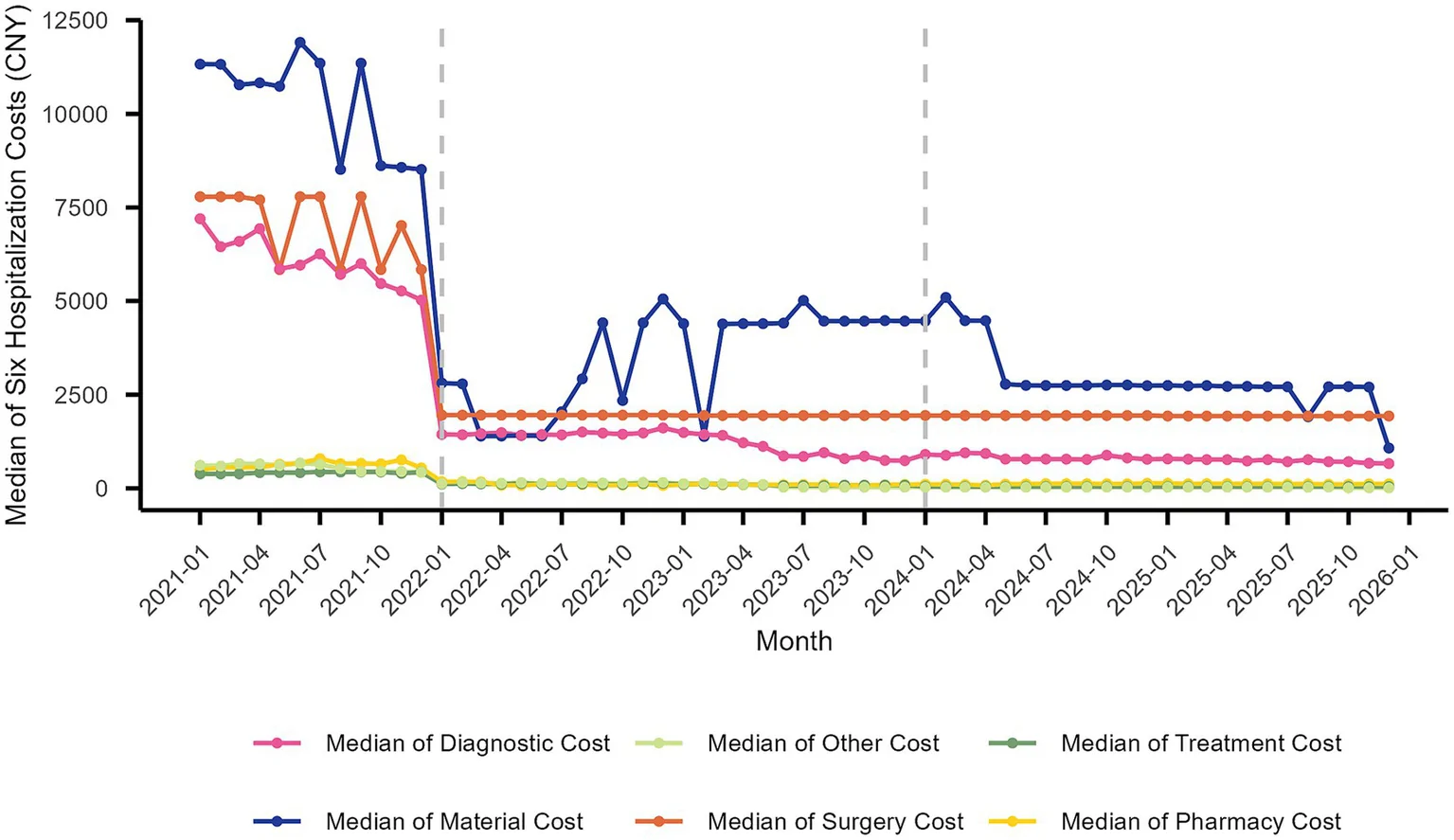
The time trend of median values for various hospitalization costs. Vertical dashed lines indicate the implementation dates of the VBP (January 2022) and DRG (January 2024) policies. Each point represents the monthly median cost for the respective category, aggregated from all individual patient records within that calendar month.

The cost indicators generally showed a downward trend across the three time periods, although some costs exhibited periodic fluctuations. From the Time Period 1 to the Time Period 3, the median total hospitalization cost decreased from 27,403.68 Chinese yuan (CNY) to 5,516.60 CNY (a decrease of 79.9%), and the median material cost decreased from 11,119.44 CNY to 2,741.10 CNY (a decrease of 75.4%) (Table 3). Following the implementation of the VBP in 2022, both the total cost and the material cost experienced a sharp decrease, but subsequently rebounded; in contrast, diagnostic cost, treatment cost, and others continued to show a downward trend. After the implementation of the DRG payment reform in 2024, the total cost and material cost returned to a downward trajectory, with the rebounding trend of material cost being effectively curbed; surgery cost remained stable, while the remaining cost categories either stabilized or fluctuated within a narrow range (Figures 1, 2). Overall, the sequential implementation of the two policies significantly reduced the various costs of inpatients, drove the progressive reduction of total hospitalization cost and material cost, and exerted a positive influence on the adjustment of the hospitalization expenditure structure.

**Table 3:** Median and interquartile range (IQR) of hospitalization costs across the three periods [M(Q_1_, Q_3_)].

Structure of cost, CNY	Time Period 1 *n* = 1,001	Time Period 2 *n* = 2,981	Time Period 3 *n* = 4,476
Total hospitalization cost	27403.68 (21193.71, 34906.76)	7214.21 (5147.17, 8266.28)	5516.60 (4497.65, 6001.51)
Pharmacy cost	633.26 (485.19, 775.47)	96.37 (78.21, 113.10)	111.21 (96.37, 122.35)
Material cost	11119.44 (8461.23, 13475.30)	4382.92 (1392.77, 5031.99)	2741.10 (1020.51, 2795.96)
Treatment cost	416.00 (327.00, 490.00)	103.31 (81.84, 128.74)	42.91 (42.62, 44.60)
Surgery cost	7787.80 (5840.85, 7787.80)	1943.06 (1943.06, 1952.81)	1943.06 (1929.58, 1943.06)
Diagnostic cost	5968.00 (4890.00, 7335.00)	1248.50 (793.41, 1505.99)	771.46 (599.60, 1072.85)
Other cost	596.24 (444.56, 770.31)	111.32 (27.64, 159.12)	28.14 (26.75, 29.53)

In 2021, 1 USD ≈ 6.4515 CNY.

### 3.3 Proportion of hospitalization costs

**Figure 3:**
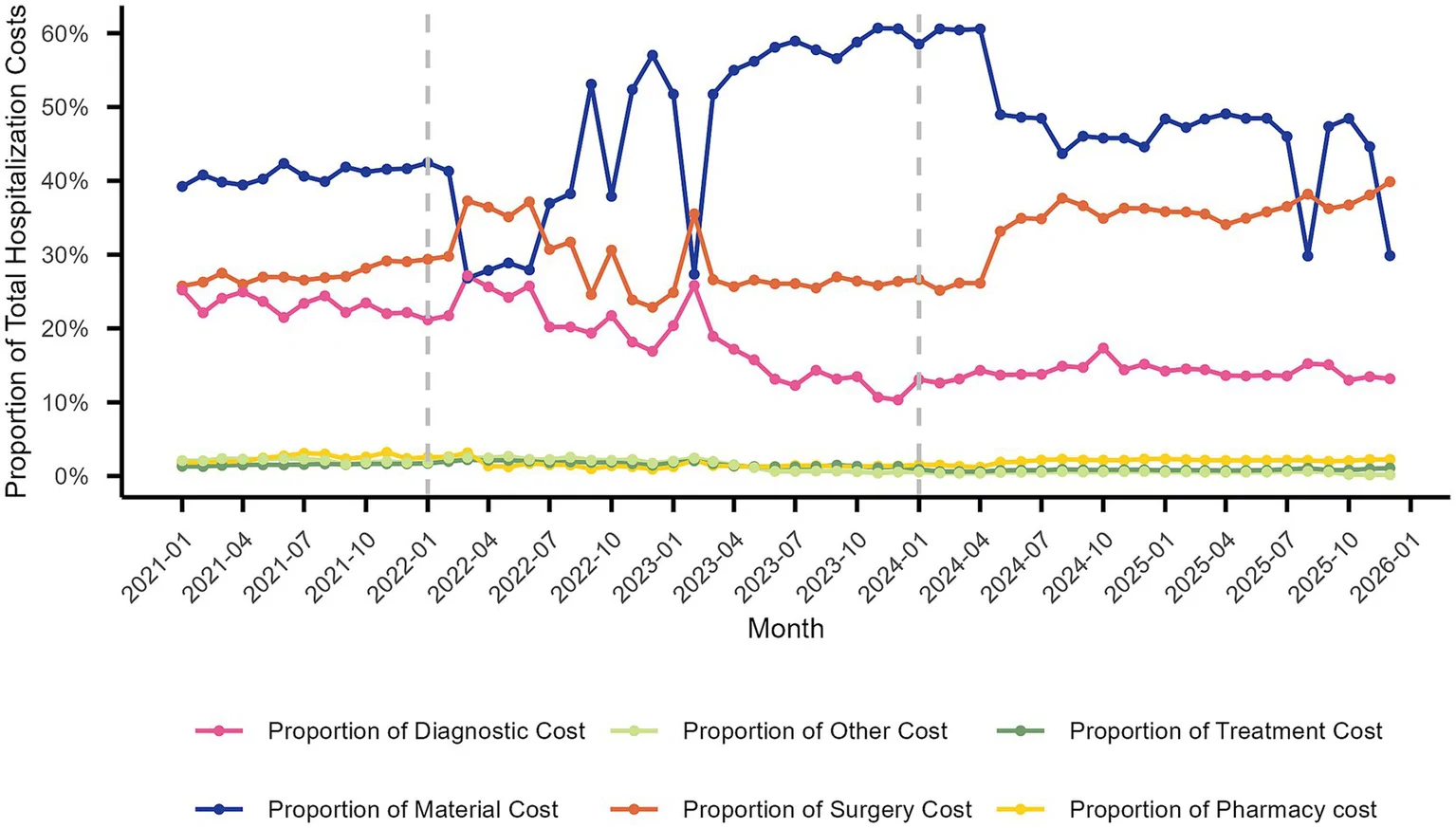
The time trend of median proportions of various hospitalization costs. Vertical dashed lines indicate the implementation dates of the VBP (January 2022) and DRG (January 2024) policies. Each point represents the monthly median proportion for the respective category, aggregated from all individual patient records within that calendar month.

From the pre-policy period to the post-VBP and pre-DRG period, and further to the post-DRG period, the proportions of each hospitalization costs category in the total cost exhibited phased changes across the three time periods (Figure 3; Table 4). In the pre-policy period, material cost accounted for the highest proportion of the total cost, at 40.91%, followed by surgery cost (27.26%) and diagnostic cost (23.36%); the proportions of pharmacy cost, treatment cost, and other cost were relatively low (all < 3%). Following the implementation of the VBP, the proportion of material cost dropped sharply and then rebounded slowly, rising from 40.91 to 51.07%; the proportion of diagnostic cost decreased from 23.36 to 17.44%, while the proportion of surgery cost remained relatively stable. After the implementation of the DRG payment policy, the proportion of material cost decreased to 48.06%, the proportion of surgery cost increased markedly to 35.35%, and the proportion of diagnostic cost further declined to 13.96%; the proportion of pharmacy cost rebounded slightly, while the proportions of treatment cost and other cost fell below 1%. Overall, the two policies drove a shift in the expenditure structure from material cost toward surgery cost; however, the proportion of material cost remained higher than the pre-policy level.

**Table 4:** Median and interquartile range (IQR) of the proportion of hospitalization costs across the three periods [M(Q_1_, Q_3_)].

Structure of cost, %	Time Period 1 *n* = 1,001	Time Period 2 *n* = 2,981	Time Period 3 *n* = 4,476
Pharmacy cost	2.37 (1.55, 2.98)	1.42 (1.05, 2.04)	2.09 (1.74, 2.48)
Material cost	40.91 (38.15, 48.08)	51.07 (27.91, 60.39)	48.06 (27.34, 51.62)
Treatment cost	1.56 (1.23, 1.85)	1.61 (1.15, 2.15)	0.81 (0.73, 1.12)
Surgery cost	27.26 (17.52, 29.12)	27.44 (23.83, 38.59)	35.35 (32.56, 44.57)
Diagnostic cost	23.36 (15.00, 26.66)	17.44 (11.47, 25.30)	13.96 (10.70, 19.68)
Other cost	2.16 (1.59, 2.83)	1.69 (0.59, 2.58)	0.52 (0.44, 0.73)

### 3.4 Results of the interrupted time series models

#### 3.4.1 Effect on each category of hospitalization costs

The effects of the two reforms on total hospitalization cost and each cost category are presented below (Table 5; Supplementary Figure S1). At the initial level before the reforms, the median of total hospitalization cost, material cost, pharmacy cost, surgery cost, treatment cost, diagnostic cost, and other cost were 30,502.80 CNY, 11,770.04 CNY, 554.63 CNY, 7,866.88 CNY, 391.51 CNY, 6,970.26 CNY, and 676.76 CNY, respectively.

**Table 5:** ITS analysis of hospitalization costs and their itemized components.

Items of cost	Total hospitalization cost	Material cost	Pharmacy cost	Surgery cost	Treatment cost	Diagnostic cost	Other cost
Method Employed	OLS + Robust SE	OLS	OLS + Robust SE	OLS + Newey-West	OLS + Newey-West	OLS + Robust SE	OLS + Newey-West

	Coefficient	*P*	Coefficient	*P*	Coefficient	*P*	Coefficient	*P*	Coefficient	*P*	Coefficient	*P*	Coefficient	*P*
*β_0_*	30502.80	<0.001	11770.04	<0.001	554.63	<0.001	7866.88	<0.001	391.51	<0.001	6970.26	<0.001	676.76	<0.001
*β_1_*	−697.60	<0.001	−263.76	<0.001	13.20	0.085	−145.33	<0.001	4.10	0.004	−165.26	<0.001	−21.10	<0.001
Reform 1														
*β_2_*	−16424.80	<0.001	−7125.91	<0.001	−570.00	<0.001	−4312.71	<0.001	−310.39	<0.001	−3464.81	<0.001	−264.05	<0.001
*β_3_*	759.98	<0.001	401.38	<0.001	−15.33	0.049	144.72	<0.001	−6.13	<0.001	130.41	<0.001	14.97	0.002
Reform 2														
*β_4_*	−1025.53	0.011	−978.00	0.048	29.44	0.003	5.95	0.200	−34.52	<0.001	44.01	0.498	−1.21	0.963
*β_5_*	−150.15	<0.001	−227.30	<0.001	2.56	0.014	−0.23	0.505	2.07	<0.001	5.90	<0.001	5.61	0.001
Adjusted-*R^2^*	0.984		0.927		0.967		0.973		0.994		0.994		0.978	

Prior to the implementation of VBP, the median of total hospitalization cost, material cost, surgery cost, diagnostic cost, and other cost decreased by 697.60 CNY (*p* < 0.001), 263.76 CNY (*p* < 0.001), 145.33 CNY (*p* < 0.001), 165.26 CNY (*p* < 0.001), and 21.10 CNY (*p* < 0.001) per month, respectively, while the median of pharmacy cost and treatment cost increased by 13.20 CNY (*p* = 0.085) and 4.10 CNY (*p* = 0.004) per month, respectively.

At the implementation of VBP, the medians of total hospitalization cost, material cost, pharmacy cost, surgery cost, treatment cost, diagnostic cost, and other cost immediately decreased by 16,424.80 CNY (*p* < 0.001), 7,125.91 CNY (*p* < 0.001), 570.00 CNY (*p* < 0.001), 4,312.71 CNY (*p* < 0.001), 310.39 CNY (*p* < 0.001), 3,464.81 CNY (*p* < 0.001), and 264.05 CNY (*p* < 0.001), respectively. All changes at the implementation of VBP were statistically significant. In the period following the implementation of VBP, compared with the pre-VBP trends, the medians of total hospitalization cost, material cost, surgery cost, diagnostic cost, and other cost increased by 759.98 CNY (*p* < 0.001), 401.38 CNY (*p* < 0.001), 144.72 CNY (*p* < 0.001), 130.41 CNY (*p* < 0.001), and 14.97 CNY (*p* = 0.002) per month, respectively, while the medians of pharmacy cost and treatment cost decreased by 6.13 CNY (*p* < 0.001) and 15.33 CNY (*p* = 0.049) per month, respectively. All trend changes after the implementation of VBP were statistically significant.

In the month when DRG was first implemented, the median of total hospitalization cost, material cost, treatment cost, and other cost decreased by 1,025.53 CNY (*p* = 0.011), 978.00 CNY (*p* = 0.048), 34.52 CNY (*p* < 0.001), and 1.21 CNY (*p* = 0.963) per month, respectively, while the median of pharmacy cost, surgery cost, and diagnostic cost increased by 29.44 CNY (*p* = 0.003), 5.95 CNY (*p* = 0.200), and 44.01 CNY (*p* = 0.498) per month, respectively. No statistically significant changes were observed in surgery cost, diagnostic cost, or other cost in the month when DRG was implemented. Compared with the pre-DRG trend, after the implementation of DRG, the median of total hospitalization cost, material cost, and surgery cost decreased by 150.15 CNY (*p* < 0.001), 227.30 CNY (*p* < 0.001), and 0.23 CNY (*p* = 0.505) per month, respectively, while the median of pharmacy cost, treatment cost, diagnostic cost, and other cost increased by 2.56 CNY (*p* = 0.014), 2.07 CNY (*p* < 0.001), 5.90 CNY (*p* < 0.001), and 5.61 CNY (*p* < 0.001) per month, respectively. No statistically significant trend changes were observed in surgery cost or treatment cost after the implementation of DRG.

Based on the model specification, the actual post-policy slopes were calculated as linear combinations of the coefficients: *β₁* + *β₃* for the post-VBP period, and *β₁* + *β₃* + *β₅* for the post-DRG period. For total hospitalization cost, the pre-VBP slope was −697.60 CNY per month; after VBP implementation, the slope reversed to +62.38 CNY per month (−697.60 + 759.98), indicating a shift from decline to increase; after DRG implementation, the slope returned to −87.77 CNY per month (+62.38–150.15), indicating a resumption of the downward trend. For material cost, the post-VBP slope was +137.62 CNY per month (−263.76 + 401.38), and the post-DRG slope was −89.68 CNY per month (+137.62–227.30).

The seven interrupted time series models demonstrated a high degree of goodness-of-fit, with all adjusted *R*^2^ values exceeding 0.92, indicating that the models were able to adequately explain the variation in the trend of each cost category. Overall, the VBP effectively reduced material cost, although a subsequent rebound was observed; the DRG payment reform successfully curbed this rebounding trend, driving the total hospitalization cost and material cost back onto a downward trajectory.

#### 3.4.2 Effect on the proportions of the hospitalization cost composition

According to the results of the interrupted time series models, the proportions of each cost category in the total cost exhibited different changing trends before and after the policy interventions (Table 6 and Supplementary Figure S2) Before the reforms, the proportions of material cost, pharmacy cost, surgery cost, treatment cost, diagnostic cost, and other cost in the total hospitalization cost were 39.71, 1.93, 25.81, 1.36, 24.19, and 2.32%, respectively. In the month when VBP was implemented, the proportion of material cost decreased significantly by 12.03% (*p* < 0.001), and the proportion of pharmacy cost decreased by 0.92% (*p* = 0.060), while the proportions of treatment cost, diagnostic cost, and other cost increased significantly. Compared with the pre-VBP trend, after the implementation of VBP, the proportion of material cost began to rebound significantly, increasing by 1.19% per month (*p* < 0.001), while the proportions of pharmacy cost, surgery cost, treatment cost, diagnostic cost, and other cost all decreased significantly (*p* < 0.05). The combined post-VBP slope (*β₁* + *β₃*) for material cost proportion was +1.37 percentage points per month (0.18 + 1.19), indicating a continued upward trend in the material cost share during this period. In the month when DRG was implemented, the proportion of material cost further decreased by 5.62% (*p* = 0.073), and the proportions of surgery cost and diagnostic cost increased by 4.49% (*p* = 0.251) and 2.36% (*p* = 0.076), respectively. Compared with the pre-DRG trend, after the implementation of DRG, the proportion of material cost continued to decrease, declining by 2.10% per month (*p* < 0.001), while the proportions of pharmacy cost, surgery cost, treatment cost, diagnostic cost, and other cost all increased to varying degrees (*p* < 0.05). The combined post-DRG slope (*β₁* + *β₃* + *β₅*) for material cost proportion was −0.73 percentage points per month (0.18 + 1.19–2.10), indicating a return to a declining trajectory for the material cost share. The goodness-of-fit of the models for each cost proportion was satisfactory (adjusted *R^2^* ranging from 0.55 to 0.91), indicating that the models adequately explained the dynamic changes in the expenditure structure.

**Table 6:** ITS analysis of the composition proportion of hospitalization costs.

Items of cost	Material cost	Pharmacy cost	Surgery cost	Treatment cost	Diagnostic cost	Other cost
Method Employed	OLS + Newey-West	OLS + Newey-West	OLS + Newey-West	OLS + Newey-West	OLS + Newey-West	OLS + Newey-West

	Coefficient (%)	*p*	Coefficient (%)	*p*	Coefficient (%)	*p*	Coefficient (%)	*p*	Coefficient (%)	*p*	Coefficient (%)	*p*
*β_0_*	39.71	<0.001	1.93	<0.001	25.81	<0.001	1.36	<0.001	24.19	<0.001	2.32	<0.001
*β_1_*	0.18	<0.001	0.09	<0.001	0.25	<0.001	0.03	<0.001	−0.17	<0.001	−0.04	0.002
Reform 1												
*β_2_*	−12.03	<0.001	−0.92	0.060	5.05	0.052	0.46	<0.001	3.92	0.010	1.03	<0.001
*β_3_*	1.19	<0.001	−0.13	<0.001	−0.63	<0.001	−0.01	<0.001	−0.44	<0.001	−0.06	0.016
Reform 2												
*β_4_*	−5.62	0.073	0.58	0.129	4.49	0.251	−0.51	<0.001	2.36	0.076	−0.01	0.988
*β_5_*	−2.10	<0.001	0.07	0.023	0.82	<0.001	0.01	<0.001	0.61	<0.001	0.09	0.028
Adjusted-*R^2^*	0.575		0.554		0.647		0.911		0.841		0.892	

### 3.5 Annual costs before and after the reforms

**Figure 4:**
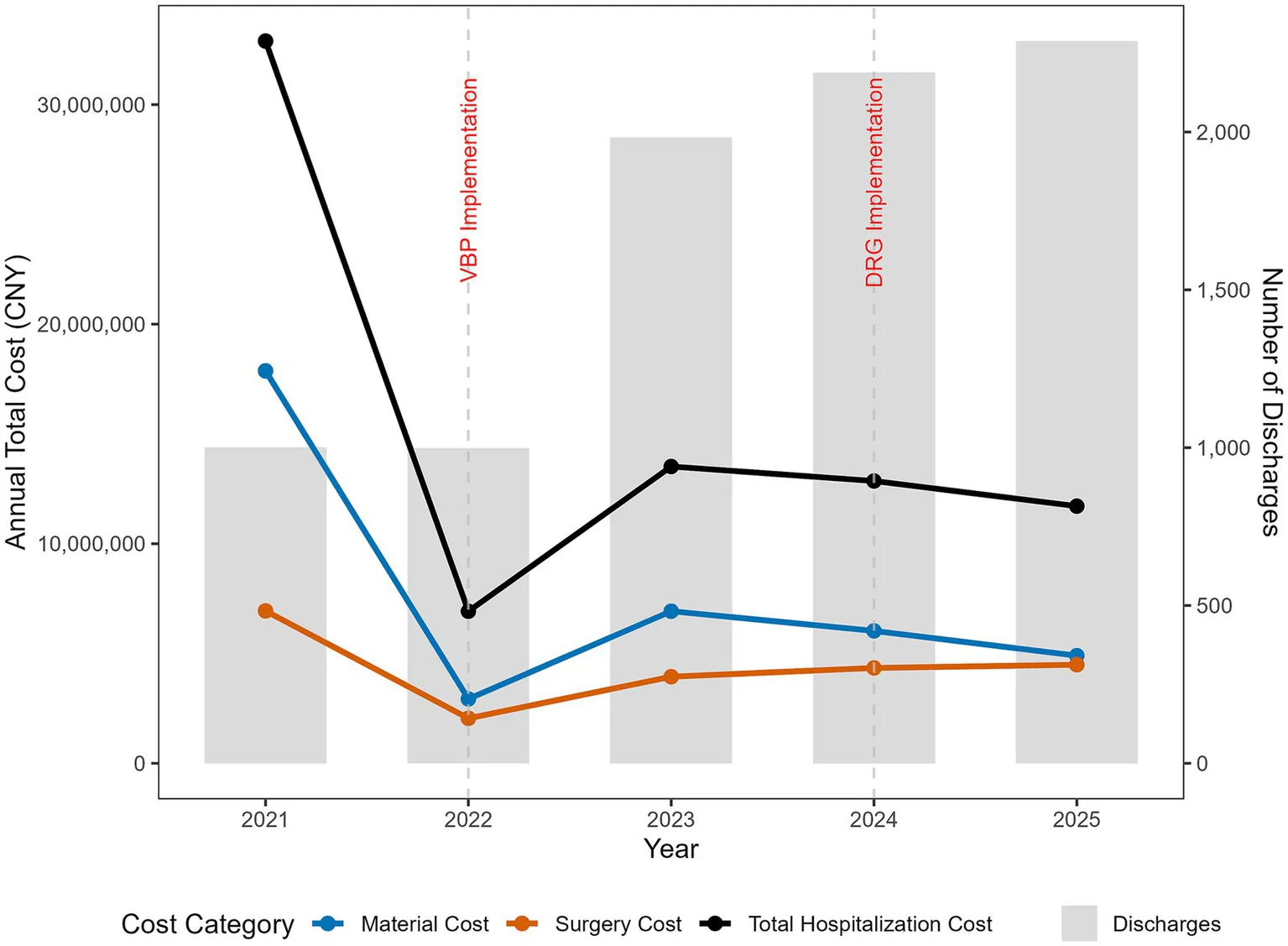
Annual trends in total costs and surgical volume for cataract surgery across three policy periods.

To capture the net financial impact of the two policies on overall hospital expenditure, we examined annual aggregate costs across the three policy periods. Figure 4 presents the annual trends in total hospitalization cost, material cost, and surgical cost from 2021 to 2025, with the annual surgical volume overlaid as a bar chart. Supplementary Table 3 summarizes the period-averaged data.

As shown in Figure 4, all three cost categories exhibited a consistent pattern: annual total costs decreased sharply from Time Period 1 to Time Period 2, but partially rebounded in Time Period 3. Specifically, the annual total hospitalization cost decreased from 32.89 million CNY in Time Period 1 to 10.22 million CNY in Time Period 2, then increased to 12.28 million CNY in Time Period 3. The annual total material cost followed a similar trajectory, declining from 17.87 million CNY to 4.93 million CNY, then rising to 5.47 million CNY. The annual total surgical cost fell from 6.94 million CNY to 3.00 million CNY, then increased to 4.42 million CNY.

This U-shaped pattern is explained by the nearly three-fold increase in surgical volume—from a yearly average of 1,001 cases in Time Period 1 to 1,491 in Time Period 2, and further to 2,238 in Time Period 3. While per-case costs declined substantially over the study period (total hospitalization cost per case: 32,861 CNY → 5,497 CNY, a reduction of 83.3%), the rapid expansion in surgical volume partially offset these per-case savings at the aggregate level.

Critically, the annual total surgical cost increased by 47.4% from Time Period 2 to Time Period 3, closely paralleling the 50.1% increase in surgical volume. However, the mean surgical cost per case declined by 71.5% (from 6,934 CNY to 1,974 CNY) over the same period. This reveals a critical tension: surgeons are handling a substantially larger clinical workload while receiving significantly less compensation per procedure—a finding with important implications for the adequacy of physician reimbursement for technical services.

## 4 Discussion

Based on real-world data of cataract patients who underwent intraocular lens implantation between 2021 and 2025 at a tertiary Grade-A hospital in western China, this study employed a multiple-phase interrupted time series model to systematically evaluate the immediate and long-term effects of the VBP for intraocular lenses and the DRG payment reform on hospitalization costs and expenditure structure. The study found that in the month when the VBP was implemented, total hospitalization cost, material cost, and surgery cost decreased significantly, and the proportion of material cost dropped sharply; however, a reversal was observed after the VBP, with total hospitalization cost and material cost reversed to a monthly increase. After the implementation of the DRG payment policy, the rebounding trend was curbed: total hospitalization cost and material cost shifted to a sustained decline, the proportion of surgery cost increased markedly, and the expenditure structure shifted from being heavily reliant on consumables toward more oriented toward surgical services. Notably, the number of patients increased nearly three-fold over the study period, suggesting that the two policies reduced the financial burden of surgery and improved patients’ access to surgical treatment.

A declining trend in total hospitalization costs was already observed in the pre-reform period (*β₁* = −697.60 CNY per month). This is consistent with anticipatory effects—hospitals may adjust practices in advance when reforms are foreseeable. The forthcoming VBP for intraocular lenses was widely publicized throughout 2021, and regional alliance-based procurement pilots had already been conducted in several provinces. These signals likely prompted hospitals to adjust clinical pathways and reduce discretionary expenditures prior to the formal policy implementation. Importantly, the ITS model controls for this pre-existing trend through the *β₁* coefficient, ensuring that the policy effect estimates represent changes beyond the underlying secular trajectory.

In the month when the VBP was implemented, total hospitalization cost, material cost, surgery cost, and all other cost categories decreased significantly, and the proportion of material cost dropped sharply by 12% (*p <* 0.001). This may be attributable to the fact that the VBP directly reduced the procurement price of intraocular lenses and associated surgical cost through the “volume-for-price” mechanism; meanwhile, hospitals also implemented operational adjustments during the initial phase of the policy, which may have contributed to the decrease in other cost categories (9, 29). However, the VBP only reduced the unit procurement price of medical consumables, without changing the fee-for-service revenue model of hospitals, and the price reduction effect brought about by the VBP did not prove to be sustainable. Sometime after the implementation of the VBP, total hospitalization cost and material cost shifted to a monthly increase, and the rebound in the proportion of material cost continued. Under the fee-for-service payment model, hospitals may have responded to the revenue losses caused by the reduced prices of medical consumables by increasing the volume of consumables used (e.g., using more auxiliary devices), switching to non-procured products with higher financial returns (e.g., premium functional intraocular lenses), or increasing the frequency of services such as examinations and laboratory tests, thereby resulting in cost reallocation (30, 31). This substitution effect has been widely documented in other settings. In the context of coronary stent procurement in China, Jian et al. (32) found that following the sharp price reduction of coronary stents, the probability of AMI patients receiving PCI with balloon—a procedure not subject to the procurement—increased from 2.2 to 6.1%, effectively offsetting some of the intended cost savings. Similarly, studies conducted after the implementation of the zero-markup drug policy have documented comparable provider-side behavioral adjustments (33). These findings collectively suggest that without concurrent payment reform, supply-side price regulation alone may merely reallocate costs rather than eliminate them, and that a single price regulation policy is unlikely to achieve sustainable cost-containment goals.

The reductions in diagnostic and surgical costs observed in the immediate post-VBP period warrant further explanation, as these categories were not directly targeted by the policy. These reductions can be attributed to several hospital-level behavioral adjustments rather than direct policy mandates. First, the decline in diagnostic costs likely reflects both the streamlining of preoperative testing protocols and, more importantly, the reconfiguration of service delivery that shifted routine examinations from inpatient to outpatient settings—a clinical practice adjustment that effectively removed these expenses from inpatient records. Second, the decrease in surgical costs requires a distinct explanation, as surgical costs and material costs are financially independent categories in China’s hospital accounting system. We propose that this immediate decline is more plausibly attributable to: (a) contemporaneous service pricing adjustments—in the context of the sharp reduction in IOL procurement prices, hospitals may have proactively adjusted surgical fee schedules as part of a broader service pricing optimization strategy; and (b) economies of scale and operational efficiency gains—with the anticipation of increased surgical volume under the new procurement framework, hospitals may have streamlined perioperative processes and accepted lower per-case margins in expectation of higher throughput. These adjustments occurred concurrently with the VBP implementation and were largely completed in the immediate post-VBP period, consistent with the immediate price-reduction effect of the policy.

However, this initial cost reduction was not sustained; total and material costs gradually rebounded in the subsequent months. The implementation of the DRG payment reform effectively curbed the aforementioned rebounding trend in costs. Following the DRG implementation, total hospitalization cost and material cost shifted to a sustained monthly decline, the proportion of material cost decreased significantly, while the proportion of surgery cost increased markedly. The underlying mechanism is as follows: the DRG-based prospective payment system transforms medical consumables, pharmaceuticals, and other items from revenue sources into cost centers for hospitals (34), motivating them to proactively control all variable costs within the fixed payment standard. Under this constraint, continued reliance on increasing the volume of consumables would only reduce hospital revenues, instead encouraging hospitals to select centrally procured consumables with higher cost-effectiveness and optimize their clinical pathways. Meanwhile, DRG payment encourages hospitals to reallocate cost reductions toward services that reflect the value of technical labor, such as surgery and treatment, which explains why the proportion of surgery cost increased markedly after the DRG payment reform while the proportion of material cost continued to decline (35). This mechanism of “curbing inappropriate incentives while promoting value-based services” represents the very core of the synergistic effect generated by the VBP and DRG payment policies, facilitating the transition from “reliance on consumables for revenue” to “revenue generation from professional services” (12). However, the interpretation of this structural shift requires caution, as discussed below.

After DRG implementation, the median proportion of surgical costs increased from 27.44 to 35.35%, while the median of surgical fee remained unchanged. This increase in proportion was driven primarily by the denominator effect—the sharp decline in the median values of other cost components, particularly material costs and diagnostic costs—rather than by an absolute rise in surgical fees. This represents a structural shift in the cost composition toward technical services at the relative level, consistent with the policy goal of reducing consumable dependence and increasing the share of service-based expenditures. However, this relative structural shift does not imply an absolute improvement in physician compensation for surgical services. While the median surgical fee per case remained stable, the annual analysis using mean per-case costs—which is necessary for computing total surgical revenue (total = mean × volume)—revealed that per-case surgical revenue in constant prices decreased over the study period. This indicates that the increase in surgical volume was accompanied by a decline in real surgical income per procedure, and that the growth in total surgical revenue was driven predominantly by volume expansion rather than by improved unit compensation.

This study identified a pattern in which hospitalization costs briefly declined and then rebounded following the implementation of the VBP, whereas after the DRG payment implementation, costs declined continuously and the expenditure structure shifted. This finding both echoes and extends previous research on the effects of single policies. First, the phenomenon of post-VBP cost rebound and a rising proportion of material cost is consistent with the cost reallocation documented after the VBP of coronary stents and the implementation of the zero-markup drug policy (33), suggesting that a reduction in the unit price of medical consumables, in the absence of payment method constraints, may trigger provider-side volume compensatory behaviors (36). However, in contrast to these studies that focus exclusively on the effects of VBP alone, this study further observed that the rebounding trend was reversed following the introduction of the DRG prospective payment system, thereby providing empirical evidence for the necessity of combining “price control with payment method reform.” Second, previous studies on the effects of DRG payment have predominantly concentrated on the reduction of total costs or average length of stay (16), with less attention paid to changes in the internal composition of costs. This study found that after the DRG implementation, the proportion of surgery cost increased markedly while the proportion of material cost continued to decline, revealing a trajectory of shifting toward technical services at the relative level. Such structural shift has rarely been documented in studies focused on DRG alone, suggesting that the price reduction baseline established by the VBP opened up fiscal space for enhancing the value of technical labor under the DRG system. Three novel contributions emerge from this comparison: (1) we provide the first empirical evidence on the sequential “rebound-correction” pattern in the same patient population; (2) we show that structural optimization does not necessarily imply absolute improvement in physician compensation—a distinction often overlooked in single-policy studies; and (3) we show that the synergistic effect of VBP and DRG is not merely additive but sequential, with DRG correcting the rebound effects of VBP. The multi-phase ITS design was essential for disentangling these sequential effects, a capability that single-phase designs lack.

Moreover, payment reforms for cataract surgery in developed countries—such as multi-tiered bundled payment models in the United States and day surgery DRG systems in several European countries-have largely relied on a single prospective payment system (37, 38), with limited involvement of VBP of high-value medical consumables. Our findings suggest that the “VBP plus DRG” policy combination may generate a distinct synergistic effect in controlling costs for high-value medical consumables and restructuring the expenditure structure, and may thus offer lessons for low- and middle-income countries facing similar challenges of population aging and rising healthcare costs (39).

Taken together, these findings suggest that the cost rebound observed when the VBP was implemented alone contrasts with the sustained decline achieved after the introduction of the DRG reform, thereby providing evidence for the necessity and the effectiveness of combining price control with payment method reform. The synergistic effect of the VBP and DRG payment policies can be explained through the following pathways: the VBP substantially reduced the unit procurement price of medical consumables through volume-based contracting, compressing consumable costs at their source and creating fiscal space for expenditure structure adjustment; the DRG prospective payment system, in turn, reshaped the expenditure composition from the payment end, encouraging hospitals to reallocate resources toward items that reflect the value of technical labor—such as surgery services—thereby contributing to the transition from reliance on consumable revenue toward professional service income. By combining these two approaches, the policy combination resulted in cost containment while redirecting resources toward technical services, mitigating the behavioral offset that often undermines single-policy interventions (40). However, while this policy combination demonstrated significant cost-containment effects and complementary synergy, it is equally important to acknowledge the respective limitations and potential risks inherent in each policy, as no payment reform is without unintended consequences.

For the VBP policy, the primary strength lies in its ability to achieve rapid and substantial price reductions through volume-based procurement, directly alleviating the financial burden of high-value consumables on patients. However, several drawbacks warrant attention. First, substantial price reductions may encourage manufacturers to cut production costs, which could potentially affect product quality and clinical performance, although strengthened quality surveillance by regulatory agencies helps mitigate this risk. Second, VBP may lead to supply shortages or market exit of certain specialized products, particularly premium or less commonly used intraocular lenses, thereby reducing clinical choice. Third, and most relevant to our findings, VBP alone may lead to hospital-level behavioral adjustments—such as increased volume of consumables, substitution with non-procured products, or increasing other service charges—as evidenced by the post-VBP cost rebound and the rising material cost proportion observed in our study. This suggests that price reduction without payment reform may lead to cost reallocation.

For the DRG payment reform, the key advantage is its prospective payment mechanism, which transforms consumables and pharmaceuticals from revenue sources into cost centers, thereby encouraging hospitals to improve efficiency and eliminate non-essential services. This mechanism directly complements VBP by addressing the post-procurement rebound and enabling expenditure reallocation toward technical services. Nevertheless, DRG also carries inherent risks. First, fixed per-case payments may encourage risk selection, such as avoiding more severe or complex patients (patient selection) or premature discharge, which could affect care quality and access. Second, providers may engage in more detailed documentation of diagnoses to reflect higher case complexity, potentially affecting payment accuracy and system fairness. Third, as discussed above, the absolute value of professional services did not improve despite the relative structural shift. This creates a tension between structural shift and the goal of fully recognizing physicians’ labor value, suggesting that fixed DRG payments need to be paired with dynamic price adjustments for technical services. Finally, DRG-based payment may limit the adoption of innovative but more costly technologies or premium IOLs if they are not fully reflected in the reimbursement weight.

These potential drawbacks suggest the need for a robust monitoring framework accompanying the “VBP plus DRG” combination, including regular quality audits, surveillance of non-procured product usage, dynamic adjustment of DRG weights and service prices, and explicit quality-of-care indicators to ensure that cost containment does not compromise clinical outcomes.

Based on the findings of this study, we propose the following recommendations. First, the synergistic implementation of the VBP of medical consumables and the DRG payment reform should be continuously advanced to prevent the effect of a single policy from being offset by hospital behavioral adjustments. Second, the prices of medical services should be dynamically adjusted to ensure that the pricing of surgery, treatment, and other technical labor items can better reflect the value of medical personnel’s skills, thereby reducing hospitals’ reliance on revenue from medical consumables. Third, regulatory oversight of non-procured consumable usage and coding practices should be strengthened to prevent cost reallocation. Fourth, quality-of-care indicators—such as readmission rates and postoperative complications—should be incorporated into the DRG performance evaluation system to ensure that cost containment does not compromise quality. In addition, to address the tension between increased surgical volume and reduced per-procedure physician compensation revealed by our annual cost analysis, we recommend dynamic adjustment of DRG weights to better reflect the complexity and intensity of surgical services. These recommendations, while grounded in China’s context, may offer lessons for low- and middle-income countries facing similar challenges of population aging and rising healthcare costs, where the “VBP plus DRG” combination could serve as a reference for high-value medical consumable governance.

An important methodological consideration is that the ITS model itself controlled for the pre-existing downward trend in costs through the *β₁* coefficient, ensuring that the policy effect estimates (*β₂* through *β₅*) represent changes beyond the underlying secular trajectory. This is particularly relevant given that the declining trend observed in the pre-reform period (−697.60 CNY per month) was not unique to our study setting but rather reflects broader patterns of anticipatory behavior and ongoing cost-containment efforts that may be present in similar policy evaluations. The ability of the multi-phase ITS design to account for such pre-existing trends—and to disentangle the independent effects of two sequential policy interventions—represents a key methodological strength of this study, distinguishing it from simpler pre-post comparisons that cannot differentiate between secular changes and policy effects.

This study has several limitations. First, as a single-center retrospective analysis conducted in western China, the generalizability of the findings is inherently limited. Second, clinical characteristics such as disease severity and comorbidities were not incorporated into the analysis, which may have introduced residual confounding. Third, the absence of quality-of-care indicators—including postoperative visual acuity, complication rates, and reoperation rates—precludes the ability to completely rule out the risk that cost reductions may have been achieved while compromising quality. Fourth, although the ITS model controlled for secular time trends, it cannot fully exclude the potential interference of other concurrent policies or external factors during the study period. Fifth, as discussed above, our annual cost analysis revealed that surgical volume growth partially offset per-case savings, suggesting the need for future research to examine the net financial impact from the payer’s perspective. Sixth, our dataset did not contain detailed information on the specific types of intraocular lenses implanted (e.g., spherical, toric, multifocal, trifocal, EDOF), which precluded stratification by IOL category. Given the substantial price differences among these lens types, the observed changes in material costs and total hospitalization costs could be partially confounded by shifts in the case-mix of IOLs used over time. However, in our institution, IOL selection is primarily driven by patients’ ocular conditions and visual needs rather than by policy changes, and the proportional distribution of IOL types is unlikely to have changed considerably during the study period. This mitigates—though does not eliminate—the risk of systematic bias. Future studies with access to IOL-specific data should incorporate this variable to achieve more granular cost analyses. Seventh, while our quantitative analysis captures the magnitude of cost changes, it does not capture the experiential and perceptual dimensions of policy impact—such as clinicians’ perceptions of workload, compensation adequacy, and practice adaptations. Future research employing mixed methods—including surveys and semi-structured interviews with hospital administrators and frontline clinicians—would be valuable to complement our findings. Additionally, Future research could be extended to multicenter prospective studies and further explore subgroup differences across dimensions such as age, gender, and health insurance type.

## 5 Conclusion

The combination of the VBP for intraocular lenses and the DRG payment reform significantly reduced the total hospitalization cost and material cost of cataract patients, restructured the expenditure structure, and the implementation of the DRG largely prevented the cost rebound that occurred following the VBP. Specifically, the median total hospitalization cost decreased by 79.9% (from 27,403.68 CNY to 5,516.60 CNY), and the median material cost decreased by 75.4% (from 11,119.44 CNY to 2,741.10 CNY). The proportion of surgical cost increased from 27.3 to 35.3%, reflecting a relative shift toward technical service value. However, annual total cost analysis revealed that the near-tripling of surgical volume partially offset aggregate savings, and per-case surgical fees declined by 71.5%, revealing a tension between increased clinical workload and reduced physician compensation per procedure. The policy synergy plays a positive role in containing healthcare costs and promoting the return of public hospitals to their public welfare orientation; however, continuous monitoring of cost reallocation patterns and changes in quality of care remains essential. Future reforms should focus on dynamic adjustment of medical service prices and DRG weights to better reflect the value of physicians’ technical labor.

## Data Availability

The raw data supporting the conclusions of this article will be made available by the authors upon reasonable request, without undue reservation.
