# Supplementary material for: Impact of the volume-based procurement for intraocular lenses combined with the diagnosis-related group’s payment reform on hospitalization costs and expenditure structure among cataract patients: an interrupted time series analysis
Source: Front Public Health. 2026 Sep 10;14:1905462. doi: 10.3389/fpubh.2026.1905462 (PMC13600984; doi:10.3389/fpubh.2026.1905462)
Supplement: Supplementary file 1 [file Data_Sheet_1.PDF]

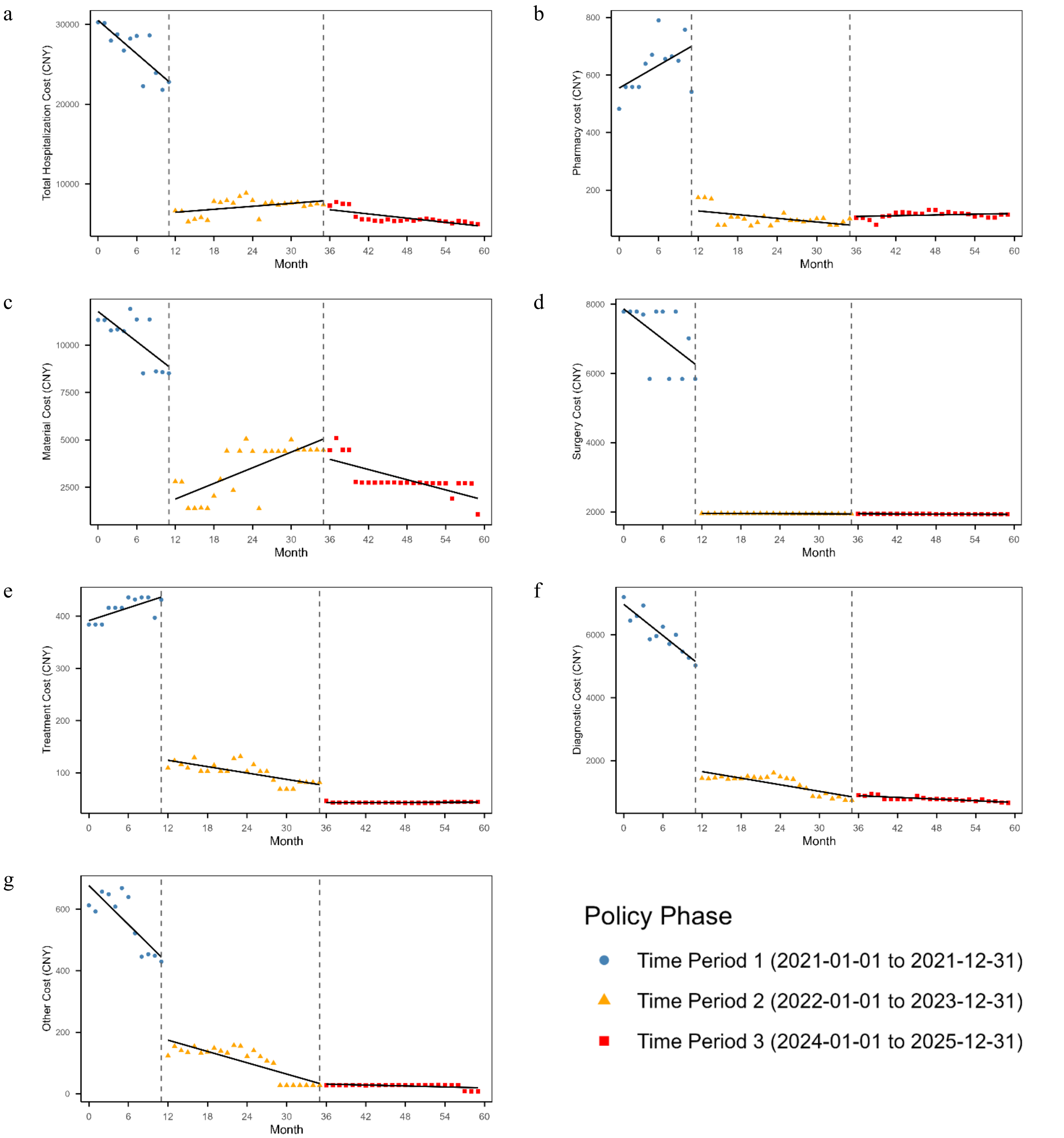

SUPPLEMENTARY FIGURE 1

Trend in median of total cost and its structure of inpatients in Cataract from 2021 to 2025.Note: Median of cost of inpatient (A)Total hospitalization cost, (B) Pharmacy cost, (C) Material cost, (D) Surgery cost, (E) Treatment cost, (F) Diagnostic cost, (G) Other cost.
