# Supplementary material for: Impact of the volume-based procurement for intraocular lenses combined with the diagnosis-related group’s payment reform on hospitalization costs and expenditure structure among cataract patients: an interrupted time series analysis
Source: Front Public Health. 2026 Sep 10;14:1905462. doi: 10.3389/fpubh.2026.1905462 (PMC13600984; doi:10.3389/fpubh.2026.1905462)
Supplement: Supplementary file 2 [file Data_Sheet_2.PDF]

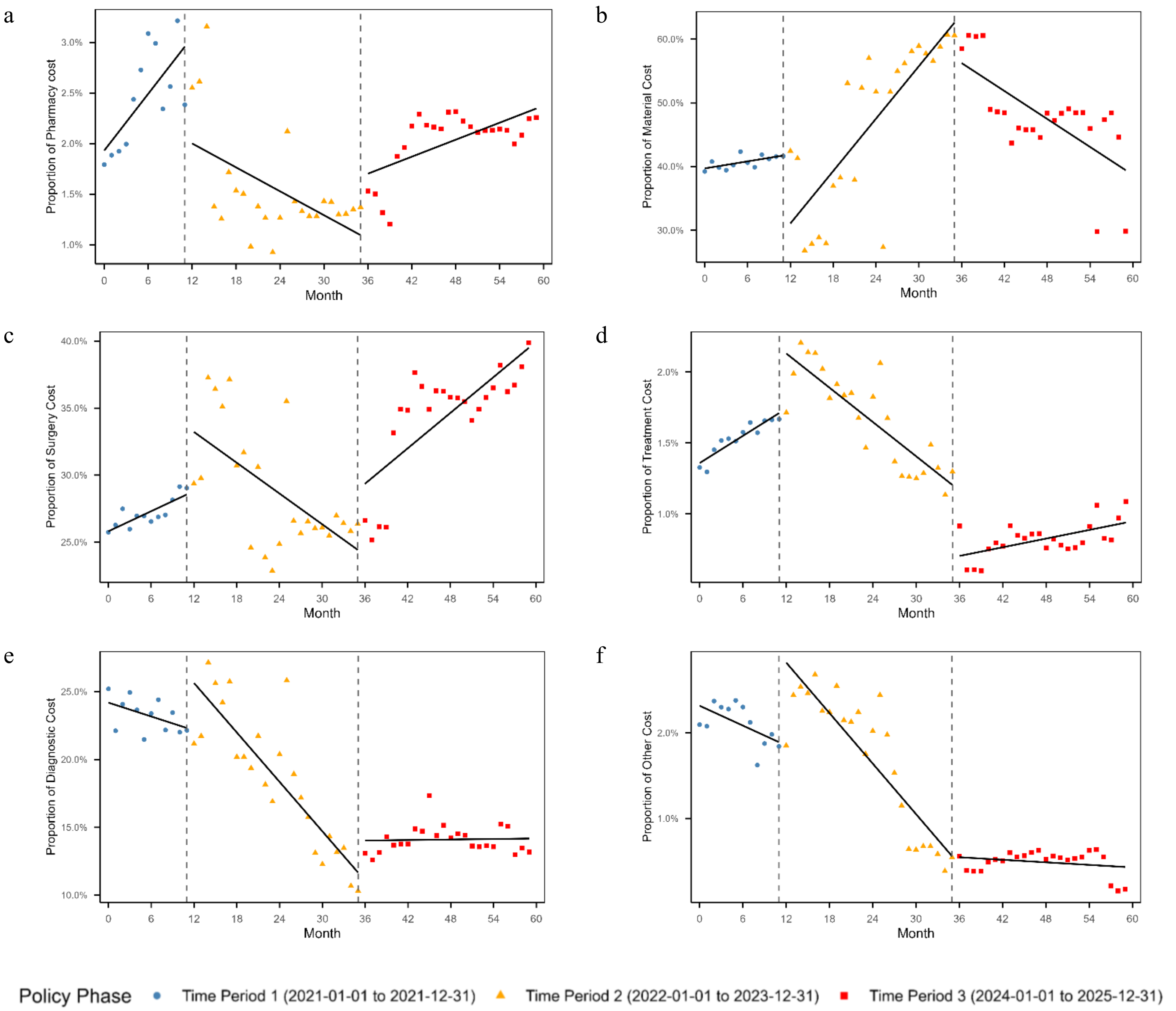

SUPPLEMENTARY FIGURE 2

Trend in proportion of total cost's compositions of inpatients in Cataract from 2021 to 2025. Note: Proportion of inpatient (A) Western medicine cost, (B) Material cost, (C) Surgery cost, (D) Treatment cost, (E) Diagnostic cost, (F) Other cost.
