# Supplementary material for: Impact of the volume-based procurement for intraocular lenses combined with the diagnosis-related group’s payment reform on hospitalization costs and expenditure structure among cataract patients: an interrupted time series analysis
Source: Front Public Health. 2026 Sep 10;14:1905462. doi: 10.3389/fpubh.2026.1905462 (PMC13600984; doi:10.3389/fpubh.2026.1905462)
Supplement: Supplementary file 3 [file Table_1.DOCX]

**Supplementary Table 1** Median and interquartile range (IQR) of hospitalization costs across the three stages $[M(Q_{1},Q_{3})]$

| Structure of cost, CNY | Time Period 1  n=985 | Time Period 2  n=2973 | Time Period 3  n=4476 |
| --- | --- | --- | --- |
| Total hospitalization cost | 27382.44(21181.47,34464.81) | 7200.98(5143.19,8251.77) | 5516.60(4497.65,6001.51) |
| Pharmacy cost | 633.26(485.19,778.84) | 96.37(78.21,113.10) | 111.21(96.37,122.35) |
| Material cost | 11119.44(8461.23,13416.55) | 4382.50(1392.77,5031.99) | 2741.10(1020.51,2795.96) |
| Treatment cost | 416.00(327.00,492.00) | 103.31(81.84,128.74) | 42.91(42.62,44.60) |
| Surgery cost | 7787.80(5840.85,7787.80) | 1943.06(1943.06,1952.81) | 1943.06(1929.58,1943.06) |
| Diagnostic cost | 5967.00(4905.00,7335.00) | 1248.50(791.42,1504.51) | 771.46（599.60,1072.85) |
| Other cost | 596.60(445.17,774.56) | 111.29(27.64,159.31) | 28.14(26.75,29.53) |

**Supplementary Table 2** Median and interquartile range (IQR) of the proportion of hospitalization costs across the three stages $[M(Q_{1},Q_{3})]$

| Structure of cost, % | Time Period 1  n=985 | Time Period 2  n=2973 | Time Period 3  n=4476 |
| --- | --- | --- | --- |
| Total hospitalization cost | 2.38(1.56,2.99) | 1.42(1.05,2.04) | 2.09(1.74,2.48) |
| Pharmacy cost | 40.83(38.08,46.23) | 50.72(27.90,60.40) | 48.06(27.34,51.62) |
| Material cost | 1.57(1.26,1.85) | 1.61(1.15,2.15) | 0.81(0.73,1.12) |
| Treatment cost | 27.27(18.31,29.13) | 27.55(23.87,38.61) | 35.35(32.56,44.57) |
| Surgery cost | 23.37(15.59,26.66) | 17.44(11.38,25.33) | 13.96(10.70,19.68) |
| Diagnostic cost | 2.17(1.59,2.86) | 1.70(0.58,2.58) | 0.52(0.44,0.73) |
| Other cost | 2.38(1.56,2.99) | 1.42(1.05,2.04) | 2.09(1.74,2.48) |
