# Supplementary material for: Impact of the volume-based procurement for intraocular lenses combined with the diagnosis-related group’s payment reform on hospitalization costs and expenditure structure among cataract patients: an interrupted time series analysis
Source: Front Public Health. 2026 Sep 10;14:1905462. doi: 10.3389/fpubh.2026.1905462 (PMC13600984; doi:10.3389/fpubh.2026.1905462)
Supplement: Supplementary file 4 [file Table_2.DOCX]

**Supplementary Table 3** Annual total hospitalization, material, and surgical costs across the three policy periods

| Period | Cases (n) (annual average) | Annual total hospitalization cost (million CNY) | Mean total hospitalization cost per case (CNY) | Annual material cost (million CNY) | Mean material cost per case (CNY) | Annual surgical cost (million CNY) | Mean surgical cost per case (CNY) |
| --- | --- | --- | --- | --- | --- | --- | --- |
| Time period 1 | 1001 | 32.89 | 32861.30 | 17.87 | 17852.90 | 6.94 | 6934.06 |
| Time period 2 | 1491 | 10.22 | 6876.78 | 4.93 | 3214.43 | 3.00 | 2020.10 |
| Time Period 3 | 2238 | 12.28 | 5497.32 | 5.47 | 2449.36 | 4.42 | 1973.50 |

Data are presented as annual averages across each period. Time Period 1 covers 2021 only; Time Period 2 covers 2022–2023; Time Period 3 covers 2024–2025. Annual total hospitalization cost, annual material cost, and annual surgical cost represent the mean annual total expenditure for each period.
